# Switching between hands in a serial reaction time task: a comparison between young and old adults

**DOI:** 10.3389/fnagi.2015.00176

**Published:** 2015-09-15

**Authors:** Maike Hoff, Sabrina Trapp, Elisabeth Kaminski, Bernhard Sehm, Christopher J. Steele, Arno Villringer, Patrick Ragert

**Affiliations:** ^1^Department of Neurology, Max Planck Institute for Human Cognitive and Brain SciencesLeipzig, Germany; ^2^Mind and Brain Institute, Charité and Humboldt UniversityBerlin, Germany; ^3^Institute for General Kinesiology and Exercise Science, Faculty of Sport Science, University of LeipzigLeipzig, Germany

**Keywords:** motor skill learning, aging, hand switch costs, bimanual serial reaction time task

## Abstract

Healthy aging is associated with a variety of functional and structural brain alterations. These age-related brain alterations have been assumed to negatively impact cognitive and motor performance. Especially important for the execution of everyday activities in older adults (OA) is the ability to perform movements that depend on both hands working together. However, bimanual coordination is typically deteriorated with increasing age. Hence, a deeper understanding of such age-related brain-behavior alterations might offer the opportunity to design future interventional studies in order to delay or even prevent the decline in cognitive and/or motor performance over the lifespan. Here, we examined to what extent the capability to acquire and maintain a novel bimanual motor skill is still preserved in healthy OA as compared to their younger peers (YA). For this purpose, we investigated performance of OA (*n* = 26) and YA (*n* = 26) in a bimanual serial reaction time task (B-SRTT), on two experimental sessions, separated by 1 week. We found that even though OA were generally slower in global response times, they showed preserved learning capabilities in the B-SRTT. However, sequence specific learning was more pronounced in YA as compared to OA. Furthermore, we found that switching between hands during B-SRTT learning trials resulted in increased response times (hand switch costs), a phenomenon that was more pronounced in OA. These hand switch costs were reduced in both groups over the time course of learning. More interestingly, there were no group differences in hand switch costs on the second training session. These results provide novel evidence that bimanual motor skill learning is capable of reducing age-related deficits in hand switch costs, a finding that might have important implications to prevent the age-related decline in sensorimotor function.

## Introduction

Healthy aging is associated with a progressive decline in cognitive and sensorimotor functions, affecting many activities of daily living (Cabeza, 2001; Seidler et al., 2010; Salthouse, 2011; Cai et al., 2014). For the motor domain, the most evident declines are a slowing in reaction time (RT; Morgan et al., 1994; Salthouse, 1996; Cuypers et al., 2013), a decreased ability to coordinate movements between limbs (Serrien et al., 2000; Heuninckx et al., 2004; Fujiyama et al., 2009; Van Impe et al., 2009; Goble et al., 2010; Solesio-Jofre et al., 2014), balance impairments (Iosa et al., 2014) and diminished accuracy in movement execution (Stewart et al., 2014). These behavioral deficits have been shown to be linked to several structural as well as functional brain alterations over the lifespan (for review, see King et al., 2013). For example, healthy aging has been shown to be associated with a continuous reduction in grey matter volume in several motor-related brain regions (Raz et al., 2003, 2005; Sowell et al., 2003). Furthermore, older adults (OA) exhibit decreased white matter volume in frontal and premotor regions (for review, see Fjell and Walhovd, 2010; Lebel et al., 2012). Apart from these reported alterations in brain structure, task-related functional alterations have also been demonstrated in OA. For example, there is evidence that OA exhibit stronger brain activation and recruit additional brain areas as compared to young adults (YA) during execution of various motor tasks (Ward and Frackowiak, 2003; Heuninckx et al., 2008; Berchicci et al., 2012). However, it is still a matter of debate whether such task-related over-activation is a supportive or maladaptive process in the aging brain. One theory is that this hyperactivity reflects additional recruitment of brain areas to compensate for declining functional efficiency, to be able to maintain similar performance levels as YA. This model is proposed as the Hemispheric Asymmetry Reduction in Older Adults (HAROLD; Cabeza, 2002). On the other hand, it is also possible that this task-related hyperactivity is a consequence of the progressive loss of brain function and reflects the inability to appropriately suppress ipsilateral activation, which results in decreased performance with increasing age (Zarahn et al., 2007; Bernard and Seidler, 2012).

Apart from these age-related alterations in brain structure and function, healthy aging is also associated with a decline of the neuromuscular system such as a reduction in muscle mass and strength, which has an impact on movement execution in everyday activities (Tucker et al., 2008). This decline is typically associated with an age-related decrease in functionality of upper limb extremities (Seidler et al., 2002). Hence, not only age-related cortical but also neuromuscular adaptations seem to be important factors influencing motor control in older individuals.

On a behavioral level, it has been shown that dexterous manipulation declines with increasing age, starting from middle age onwards (Dayanidhi and Valero-Cuevas, 2014). Interestingly, however, this decline in dexterous manipulation was not accompanied by a decline of hand strength, as measured with a pinch force device. In a similar vein, Lawrence et al. (2014) also found that the ability to perform fine dexterous hand manipulations declines with increasing age. This finding was also true for dexterous manipulations using the legs, suggesting that it is rather a systemic mechanism that occurs during the process of aging and not a limb-specific effect (Lawrence et al., 2014). However, some recent studies have suggested that this decline in function during the time course of aging may be decelerated by using specific training interventions and/or motor learning paradigms (for review, see Lustig et al., 2009; Seidler, 2010; King et al., 2013). For example Boyd et al. (2008) reported that OA show training-related improvements, but to a lesser extent as compared to YA. On the other hand, OA and YA seem to perform equally well in a visuomotor adaptation task (Roller et al., 2002). Similar performance outcomes between OA and YA have been shown in a complex juggling task (Voelcker-Rehage and Willimczik, 2006). It is, however, important to keep in mind that age-related motor impairments are highly task specific. For example, Sherback et al. (2010) demonstrated that while onset latencies in a visuomotor compensatory tracking task were comparable between young and old individuals, the age-related impairment became visible in slower corrective movements due to external perturbations.

Seidler (2006) demonstrated that OA show preserved learning capabilities but reduced motor adaptation as compared to YA. Apart from these online learning effects in aging, there is also evidence that aging is associated with a reduced ability to consolidate (offline learning) and preserve a newly acquired skill (Brown et al., 2009; Roig et al., 2014; Trewartha et al., 2014; Verneau et al., 2014). The outcome of such training interventions, however, seems to depend on various task parameters such as complexity and duration (Onushko et al., 2014), but also on cognitive functions, genetic predisposition and/or lifestyle factors in general (Cai et al., 2014).

Especially important for the execution of everyday activities is the ability to perform movements that depend on both hands working together. It has, however, previously been shown that OA show a progressive decline in bimanual motor coordination (Coxon et al., 2010; Goble et al., 2010; Heitger et al., 2013; Kiyama et al., 2014; Solesio-Jofre et al., 2014). This decline is thought to be related to functional alterations in the aging brain. For example, Heitger et al. (2013) showed that OA exhibited increased task-related functional connectivity in a widespread motor network as compared to YA. Whether or not this typically observed “overactivation” is due to compensatory mechanisms (Zimerman et al., 2014) or de-differentiation (Riecker et al., 2006) is still a matter of debate.

Furthermore, several studies indicated that corpus callosum structure and function declines with increasing age, which negatively impacts the ability of OA to perform bimanual movements (Fling et al., 2011; Fling and Seidler, 2012; Gooijers and Swinnen, 2014; Serbruyns et al., 2015). Therefore, the present study investigated differences in bimanual coordination between YA and OA to better understand how aging impacts bimanual motor sequence learning. As a model to explore bimanual motor performance, we used a bimanual serial reaction time task (B-SRTT), which was previously introduced by Trapp et al. (2012). Here, YA showed switch costs during B-SRTT performance that were reduced as a consequence of B-SRTT learning (Trapp et al., 2012). Open questions remain whether or not OA show similar behavioral effects and how B-SRTT learning and its retention is altered over the lifespan. Shedding light into this line of research will reveal further important insights into age-related changes in bimanual motor coordination. This information might be of particular relevance for future studies that aim to maintain or prolong an independent lifestyle with advanced age in daily activities requiring bimanual motor coordination. Based on previous findings, we hypothesized that OA would show: (a) generally slower bimanual response times as compared to YA; (b) reduced bimanual motor learning abilities; (c) greater costs for switching between hands within learning trials; (d) and reduced bimanual motor skill retention.

## Materials and Methods

### Participants

We compared B-SRTT performance of 26 OA (mean age: 60.69 ± 1.34 years, age range: 50–74 years, 15 female) with 26 YA (mean age: 25.65 ± 0.68 years age range: 20–35 years, 13 female) on two separate training days (TD1 and TD2). One old participant could not attend TD2, therefore, comparisons on TD2 were performed with 25 OA and 26 YA. All participants were right handed, as assessed with the Edinburgh Handedness Scale (mean LQ (laterality quotient) OA: 88.42 ± 3.29; mean LQ YA: 92.65 ± 2.65; −100 = full left handed to +100 = full right handed; Oldfield, 1971). There was no difference in LQ scores between YA and OA (independent-samples *t*-test *t*_(50)_ = −1.002, *p* = 0.321).

A trained physician performed a detailed neurological examination in all participants prior to participation. This examination included a short review of the individual medical history (anamnesis), the assessment of muscle strength and tone, gait and posture. Furthermore, we assessed the function of the sensory system by provoking sensations of fine touch and pain. The cerebellar functioning was assessed by testing for dysmetria, dysdiadochokinesis, ataxia and intention tremor. The cranial nerves were assessed as well as the deep tendon reflexes including biceps and triceps tendon, knee tendon, ankle jerk and Babinski sign. None of the participants showed any signs of neurological disease and all were free of central acting medication. All participants gave written informed consent before participation. The study was approved by the local ethics committee of the University of Leipzig and performed according to the declaration of Helsinki.

### Study Design

**Figure 1:**
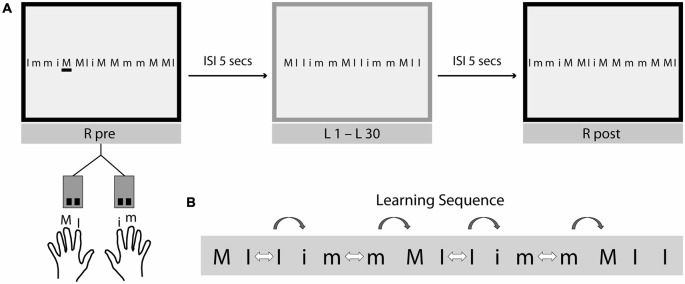
**Experimental setup and design. (A)** Bimanual Serial Reaction Time Task (B-SRTT). The task was to perform the B-SRTT on two training days (TD1 and TD2), which were separated by at least 1 week. Participants were presented with a learning sequence consisting of a 15-letter array on a computer screen, with the letters m and i. Capital letters (I, M) correspond to button presses of left index (I)- and middle finger (M) respectively and small typed letters (i, m) correspond to button presses of the right hand index (i)- and middle finger (m). The whole sequence was visible at once and the task was self-paced (=explicit motor learning task). Participants were asked to press the corresponding keys on a keyboard as fast and as accurate as possible. No time-limit was given during sequence presentation. The experiment started with the presentation of a random sequence (R pre), afterwards 30 repetitions of the learning sequence (L1–L30) were presented, followed by another presentation of the random sequence (R post). To avoid muscle fatigue, the interstimulus interval (ISI) between sequence presentations was 5 s. Each trial was followed by visual feedback regarding response times and the number of errors that participants made during the previous trial. **(B)** To investigate the effects of switching between-hands compared to switches within-hands (hand switch costs), there were 10 within-hand switches (white arrows) and 4 between-hand switches (grey arrows) implemented in the learning sequence. In order to keep a constant number of switches, only within-hand switches immediately before a between-hand switch were used for the analysis of hand switch costs.

The task was to perform a B-SRTT on two training days (TD1 and TD2), which were separated by at least 1 week. We used the same B-SRTT as previously introduced by Trapp et al. (2012). In brief, participants were presented with a learning sequence consisting of a 15-letter array on a computer screen, with the letters I and M. The stimuli were either capital letters (I, M), corresponding to button presses of left index (I)- and middle finger (M) respectively, or small typed letters (i, m) corresponding to button presses of the right hand index (i)- and middle finger (m). The 15-letter sequence was visible over the duration of the trial and the task was self-paced. The task was to press the corresponding keys on a keyboard as quickly and accurately as possible. There was no time-limit for completion. Participants performed 30 repetitions of the learning sequence (L1–L30; M I I i m m M I I i m m M I I), which were preceded (R pre) and followed (R post) by a single random sequence (R; I m m i M M I i M M m m M M I). To investigate the effects of switching between hands compared to switches within hands, there were 10 within-hand switches and four between-hand switches implemented in the learning sequence. In order to avoid muscle fatigue, the interstimulus interval (ISI) between sequence presentations was 5 s. Each trial was followed by visual feedback that provided response times and the number of errors that participants made during the previous trial. For task-familiarization, participants completed five practice trials of a different sequence (I I I M M M i i i m m m i I m) before starting the experiment. Please see Figure 1 for a description of the experimental setup.

## Behavioral Measurements

### Global B-SRTT Learning

To assess global learning, we calculated B-SRTT RTs for each sequence and calculated the improvement from L1–L30 (expressed as time to complete [s]). Sequence specific learning was assessed by computing the difference of the percentage improvement between R post and L30.

### Hand Switch Costs

The primary aim of the present study was to investigate potential differences between OA and YA regarding the effect of switching between hands as compared to within-hand switches during B-SRTT learning trials. Therefore, RTs for each individual key press for each trial of the learning sequence (L1 to L30) was also analyzed. To reduce individual inter-trial variability, RTs for individual button presses were averaged across six repetitions of the learning sequence and across participants within each group, resulting in five time bins for the learning sequence (see Figure 1B).

Since button presses for between-hand switches might also be influenced by the RT for the previous within-hand switch (e.g., I, I), we used the following formula to compute hand switch costs:

$$\text{Hand Switch Costs}=\;|({\text{KP}}_{\text{bw}}-{\text{KP}}_{\text{aw}})-({\text{KP}}_{\text{bb}}-{\text{KP}}_{\text{ab}})|$$

KP (reaction time for keypress), KP_bw_ (KP before within-hand switch), KP_aw_ (KP after within-hand switch), KP_bb_ (KP before between-hand switch), KP_ab_ (KP after between-hand switch).

Since there were ten within-hand switches and only four between-hand switches, we only used the within-hand responses immediately before a between-hand switch for this calculation in order to match for the number of between-hand switches. Hand switch costs were then defined as the absolute difference in RT for within-hand switches compared to between-hand switches. To account for slower RTs of OA as compared to YA, hand switch costs were subsequently normalized to the first time bin for each individual. Retention of hand switch costs was calculated as the difference in the amount of hand switch costs from the end of TD1 to the beginning of TD2 (Bin 5 TD1 − Bin 1 TD2) and compared between groups.

## Statistical Analysis

Data analysis was performed using the Statistical Software package for Social Sciences (IBM SPSS) version 22. In order to compare global B-SRTT learning, we performed two repeated measures analysis of variance (ANOVA-RM) separately for TD1 and TD2 with factors SEQUENCE REPETITION (L1–30) and GROUP (OA vs. YA). Subsequently, sequence specific B-SRTT learning was compared between the two groups by using a paired samples *t*-test.

Two separate ANOVA-RM with factor BIN (Bin 1 to Bin 5) and GROUP (OA vs. YA) were performed to test for alterations in hand switch costs over the time course of B-SRTT learning between the two groups on TD1 and TD2. Retention was assessed by using an independent-samples *t*-test that compared the difference in hand switch costs from TD1 to TD2 (Bin 5 TD1-Bin 1 TD2) between OA and YA.

If necessary, data was corrected for sphericity using Greenhouse-Geisser correction. Independent-samples *t*-tests were used for *post hoc* comparisons between groups and paired-samples *t*-tests were used for *post hoc* within-group comparisons. The statistical significance level was set at *p* < 0.05 and a Bonferroni correction was used to account for multiple comparisons.

## Results

### Global B-SRTT Learning

**Figure 2:**
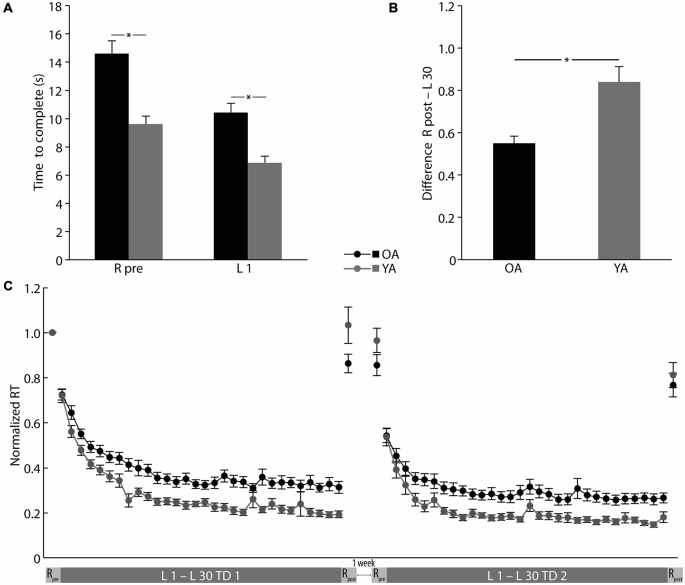
**Global and sequence specific B-SRTT learning. (A)** Initial B-SRTT performance differences between younger (YA) and older (OA) adults. **(B)** Sequence-specific B-SRTT performance on training day (TD) 1. **(C)** B-SRTT performance for TD1 and TD2 (separated by at least 1 week). For details see text. RT, Response time; R, Random sequence; L1–L30, Learning sequence 1–30. Error bars represent mean ± standard error of the mean, *p < 0.05.

OA showed significantly slower B-SRTT response times than YA already at the beginning of training (R pre OA: 14.61 ± 0.92 s, YA: 9.61 ± 0.56 s; independent samples *t*-test: *t*_(41.557)_ = 4.651, *p* = 0.000; L1 OA: 10.44 ± 0.66 s, YA: 6.87 ± 0.48 s, independent-samples *t*-test: *t*_(50)_ = 4.387, *p* = 0.000; see Figure 2A). Hence, all further analyses were performed on normalized data (normalized to the performance of the R pre) to rule out possible confounding effects of the generally slower RTs in OA on global learning performance. Over the time course of B-SRTT learning, both groups showed a significant reduction of RTs on TD1 (ANOVA-RM TIME (L1–L30) *F*_(8.186,409.323)_ = 78.591, *p* = 0.000). Interestingly, there was no difference in learning rate between groups (ANOVA-RM TIME (L1–L30) × GROUP (OA vs. YA) *F*_(8.186,409.323)_ = 1.410, *p* = 0.189), though OA had significantly slower RTs than YA (ANOVA-RM GROUP *F*_(1,50)_ = 18.205, *p* = 0.000; see Figure 2C).

Sequence-specific improvement was, however, more pronounced in YA as compared to OA (Difference R post—L30 OA: 54.97 ± 3.49%, YA: 83.96 ± 7.24%, independent-samples *t*-test *t*_(36.014)_ = −3.607, *p* = 0.001; see Figure 2B).

Initial performance (L1) on TD2 did not differ between groups (L1 TD2 OA: 0.54 ± 0.03, YA: 0.54 ± 0.04, independent-samples *t*-test: *t*_(49)_ = 0.134, *p* = 0.894). Both groups showed further reductions in global RTs on TD2 (ANOVA-RM TIME (L1–L30 TD2) *F*_(6.172,302.448)_ = 42.351, *p* = 0.000), with, again, slower RTs in OA (ANOVA-RM GROUP (OA vs. YA) *F*_(1,49)_ = 12.855, *p* = 0.001), but no difference in learning rate (ANOVA-RM TIME (L1–L30 TD2) × GROUP (OA vs. YA) *F*_(6.172,302.448)_ = 1.151, *p* = 0.332; see Figure 2C).

### B-SRTT: Hand Switch Costs

**Figure 3:**
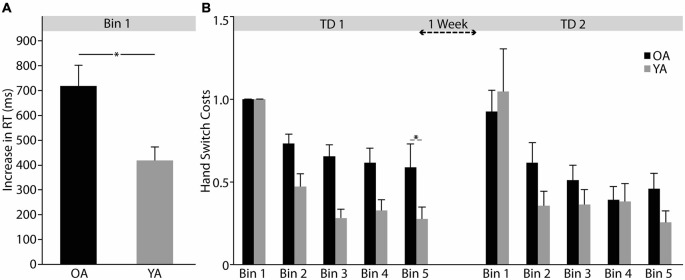
**Hand switch costs. (A)** Differences in initial hand switch costs (Bin 1) between younger (YA) and older (OA) adults. **(B)** Learning-related changes in hand switch costs for training day 1 and 2 (TD1 and TD2). For details see text. Response time (RT); One Bin consists of averaged data from six repetitions of the learning sequence. Error bars represent mean ± standard error of the mean, *p < 0.05.

In Bin 1, OA showed significantly greater hand switch costs than YA (average of learning sequences 1–6) on TD1 (Bin 1 OA: 719.01 ± 83.56 ms, YA: 418.81 ± 53.94 ms, independent-samples *t*-test: *t*_(42.755)_ = 3.019, *p* = 0.004; see Figure 3A). Hence, all further analyses steps were performed on data that was normalized to the first Bin. Both groups showed learning-related reductions in hand switch costs on TD1 (ANOVA-RM TIME (Bin 2–5) *F*_(2.242,112.079)_ = 3.042, *p* = 0.046). Interestingly, there was no difference in learning-related hand switch cost reduction between groups (ANOVA-RM TIME (Bin 2–5) × GROUP (OA vs. YA) *F*_(2.242,112.079)_ = 0.330, *p* = 0.744; see Figure 3B), indicating preserved learning capabilities in OA.

Retention of hand switch costs was not significantly different between OA and YA (Bin 5 TD1- Bin 1 TD2 OA: 0.32 ± 0.13, YA: 0.77 ± 0.26; independent-samples *t*-test *t*_(49)_ = −1.512, *p* = 0.137). On TD2, both groups showed significant reductions in hand switch costs with no difference between groups (ANOVA-RM TIME (Bin 1-Bin 5 TD2), *F*_(1.740,85.262)_ = 21.427, *p* = 0.000; TIME (Bin 1-Bin 5) × GROUP (OA vs. YA) *F*_(1.740,85.262)_ = 1.895, *p* = 0.162) and similarly, no overall difference in hand switch costs (ANOVA-RM GROUP (OA vs. YA) *F*_(1,49)_ = 0.479, *p* = 0.492; see Figure 3B).

## Discussion

The aim of the present study was to investigate potential differences in bimanual motor coordination and learning capabilities between young and old adults. In line with previous studies, we found that although OA were generally slower in global response times, they showed similar improvements over the time course of B-SRTT learning as compared to YA. However, sequence specific learning was more pronounced in YA than in OA. Even though initial hand switch costs were higher in OA, B-SRTT learning resulted in pronounced reductions of hand switch costs in both groups. Even more interesting was the finding that there were no differences in hand switch costs between YA and OA during TD2 (performed 1 week after TD1). These results provide novel evidence that bimanual motor skill learning is capable of reducing age-related deficits in hand switch costs, a finding that might have important implications for treatment strategies that aim to prevent/decelerate the age-related decline in sensorimotor function.

It has previously been shown that aging is associated with a decline in motor performance (Cai et al., 2014), that is often associated with generally slower movements (Morgan et al., 1994; Salthouse, 1996; Cuypers et al., 2013). The general slowing of movements in OA observed in e.g., reaction time tasks, has, however, mainly been attributed to decreased function in working memory and central processing (Crossley and Hiscock, 1992; Briggs et al., 1999). However, in the present study, we were interested in the general ability of OA to perform and learn new bimanual hand movement sequences, without the confounding influence of a general difference in movement speed. Interestingly, when we normalized the data to account for initial performance differences between groups, OA showed a similar learning curve as compared to YA on B-SRTT learning, indicating preserved learning capabilities in a bimanual hand motor task in OA. This finding is in accordance with several previous studies on procedural motor skill learning (Howard and Howard, 1992; Daselaar et al., 2003; Shea et al., 2006; Brown et al., 2009). An age-related slowing in response times has also been described in a previous study by Sherback et al. (2010). They found no difference in response onset latency but in movement execution when comparing YA and OA. However, using our study design we can obviously not disentangle movement onset and execution latencies since participants had to perform button presses in a self-paced manner.

Even more interesting was the finding that OA showed reduced sequence specific B-SRTT learning. Hence, OA show preserved bimanual hand coordination but a decline in sequence-specific learning capabilities. Interestingly, on the first sight this result is inconsistent with a recent study by Bhakuni and Mutha (2015) using a similar B-SRTT task. In that study, no differences in global B-SRTT and sequence-specific learning were found between YA and OA, which could be explained by different methodological approaches in both studies: first of all, while the present study used an explicit approach of the B-SRTT, Bhakuni and Mutha (2015) performed an implicit version. In fact, Verneau et al. (2014) reported that the process of aging preserves the capacity for implicit motor skill learning, while explicit skill learning declines in healthy aging. This notion might explain the aforementioned difference in the results. Also other task-related factors might have contributed to the differences: while the task in our study was self-paced, with the whole learning sequence visible at once, Bhakuni and Mutha (2015) used a paced task where stimuli occur after each other with a predefined tempo (implicit learning). Furthermore, Bhakuni and Mutha (2015) used a shorter learning sequence (12 digits) as compared to the sequence that we presented (15 digits) The reason why we chose a 15 digits B-SRTT sequence was motivated by the fact that we wanted to have similar task conditions as compared to our previous study (Trapp et al., 2012).

Previous work from our group showed increased RTs for between-hand switches in YA and a reduction of hand switch costs as a consequence of B-SRTT learning (Trapp et al., 2012). Trapp et al. (2012) hypothesized that switch costs are a result of a response conflict between homologous fingers when participants had to switch between hands, a phenomenon most likely related to interhemispheric inhibition between primary motor cortices (M1s). Although not explicitly tested by Trapp et al. (2012), alterations in interhemispheric inhibition as a consequence of learning (Camus et al., 2009) have been hypothesized to contribute to the observed reduction in hand switch costs. Here, we showed that (A) switch costs are modulated by age and (B) OA as well as YA showed reductions in switch costs by B-SRTT learning.

Since OA have been found to exhibit an altered interhemispheric as well as intracortical inhibition in primary motor cortices (Talelli et al., 2008; Fling et al., 2012), we propose that this disinhibition within and between M1s might result in more pronounced hand switch costs in OA as compared to YA. In the same vein, Coppi et al. (2014) showed, that OA exhibit a decrease in interhemispheric interaction as measured with transcranial magnetic stimulation (TMS), as well as more mirror symmetrical movements as compared to YA. These electrophysiological findings were also accompanied by a decline in fine motor skills in OA (Coppi et al., 2014). Other evidence for disturbed inhibitory motor control in OA comes from Coxon et al. (2012) who showed that the ability to suppress a motor response in a go/no go task is significantly reduced in OA as compared to YA. This reduced inhibitory control was associated with age-related alterations in specific task-related brain regions (Coxon et al., 2012). Even though previous reports show impaired inhibitory control and age-related functional alterations, we here provide novel evidence that age-related impairments in hand switch costs are not irreversible. In fact, OA showed similar learning-related reductions in hand switch costs and switch costs were finally not different between groups at the end of TD2.

Our findings regarding hand switch costs, however, are in contrast to Bhakuni and Mutha (2015). While Bhakuni and Mutha showed no learning-related reductions in hand switch costs, neither in YA nor in OA. We believe that differences in switch cost analyses might account for these divergent results. In the present study, we used new analyses steps to calculate hand switch costs. We assumed that between-hand switches are also influenced by the RT of the previous within-hand switch. This assumption is in accordance with findings of Bhakuni and Mutha (2015), who reported switch costs occurred not only for between-hand switches, but also for some within-hand switches. Therefore, our new analysis approach that takes the RT for the previous within-hand switch into account, might be more applicable to investigate the direct effects of switching between hands.

An interesting result of our study was that the retention of learning-related hand switch cost reductions revealed no age-dependent effects. Previous research has indicated that aging is associated with a loss of motor memory consolidation (Shea et al., 2006; Spencer et al., 2007). However, when participants performed the B-SRTT on TD2, we could not find differences in individual learning-rate (switch costs) between OA and YA.

In summary, we could show that (A) OA showed slower response times but (B) preserved B-SRTT learning in hand switch costs. Our finding has important implications for interventional approaches that aim to decelerate/prevent age-related declines in motor abilities. Since, we only used behavioral assessments, we can only speculate about the underlying neural mechanisms that might modulate the findings of the present study. Future neuroimaging studies should be performed to investigate the underlying neurophysiological mechanisms of age-related changes in hand switch costs as a consequence of bimanual motor skill learning.

## Funding

This research was supported by the Max Planck Society. EK was supported by the FAZIT-Stiftung. The funders had no role in study design, data collection and analysis, decision to publish, or preparation of the manuscript.

## Conflict of Interest Statement

The authors declare that the research was conducted in the absence of any commercial or financial relationships that could be construed as a potential conflict of interest.
